# Based on a Self-Feeder Layer, a Novel 3D Culture Model of Human ADSCs Facilitates Trans-Differentiation of the Spheroid Cells into Neural Progenitor-Like Cells Using siEID3 with a Laminin/Poly-d-lysine Matrix

**DOI:** 10.3390/cells10030493

**Published:** 2021-02-25

**Authors:** Liang Luo, Wei Zhang, Wenjin Chen, Xiaojun Fu, Xujie Wang, Ruxiang Xu, Dahai Hu

**Affiliations:** 1 Department of Burns and Cutaneous Surgery, Xijing Hospital, the Fourth Military Medical University, Xi’an 710003, China; doctorzhw@163.com (W.Z.); wangxujiecpu@163.com (X.W.); 2 Stem Cell Research Center, Neurosurgery Institute of PLA Army, Beijing 100700, China; uscyxychenwj@126.com (W.C.); fuxiaojun880205@163.com (X.F.); 3 Department of Plastics and Aesthetic Surgery, the First Affiliated Hospital of Xi’an Medical University, Xi’an 710077, China; 4 The Second School of Clinical Medicine, Southern Medical University, Guangzhou 510000, China; 5 Bayi Brain Hospital, General Hospital of PLA Army, Beijing 100700, China; 6 The Department of Neurosurgery, Sichuan Academy of Medical Science and Sichuan Provincial People’s Hospital, Chengdu 610072, China

**Keywords:** adipose stem cells, neural stem cells (NSCs), EP300-interacting inhibitor of differentiation 1 (EID1), mesenchymal stem cells (MSCs), reprogrammed

## Abstract

Human adipose-derived stromal cells (ADSCs) are receiving unprecedented attention as a potential cellular source for regenerative medicine-based therapies against various diseases and conditions. However, there still have significant issues concerning the translational development of ADSC-based therapies, such as its heterogeneity and being prone to aging. We developed a new simple and economical 3D semi-suspended expansion method in which 3D spheroids reside on an ADSC-derived self-feeder cell layer, producing cells with increased population homogeneity and strong stemness and ensuring that the proliferation and differentiation potency of the cells does not become notably reduced after at least ten passages in culture. To check the potential application of the 3D ADSC spheroids, we discovered that the combination of siEID3, which is a small interfering RNA of EP300 inhibitor of differentiation 3 (EID3), and laminin/poly-d-lysine matrix can rapidly result in trans-differentiation of the 3D spheroid cells to neural progenitor-like cells (NPLCs) in approximately 9 days in vitro. This approach provides a multidisciplinary tool for stem cell research and production in mesenchymal stem cell-related fields.

## 1. Introduction

Human adipose-derived stromal/stem cells (ADSCs) are isolated from the “stromal-vascular fraction” (SVF) of subcutaneous adipose tissue [1]. Due to these cells’ ready availability, large number, and abundant sources in the body, the number of studies on and applications of ADSCs have increased dramatically in recent years, and ADSCs had been extensively used in regenerative medicine mainly due to their paracrine effect, immunomodulatory functions and, differentiation capacity [2]. It is precisely because of these numerous successful applications that clinical trials have also hugely increased in number in recent years [3]. However, ADSCs exhibit high heterogeneity, which probably mainly results from various factors including donor age, different organ, isolation procedures, or culture methods [4], which affect the features of ADSCs, such as proliferative capacity, differentiation potential, aging process, immunophenotype, and secretory ability [4,5]. For this reason, the application of ADSCs for clinical treatment has been greatly restricted [6], and more efficient solutions are needed.

To address these problems, multiple solutions for ADSCs have been developed, such as low-density culture, single-cell cloning culture, flow cytometry sorting, base-specific extracellular microenvironment (ECM) matrix culture, and 3D culture [7,8,9]. Indeed, 3D culture is an effective approach based on the development of a monolayer culture system and, to a certain extent, has the characteristics of an in vivo animal model [10]. Compared to the monolayer method, 3D culture creates a microenvironment in which cells grow in three dimensions or interact with the surrounding environment [11]; furthermore, relevant ADSC communication with adjacent cells increases, readily resulting in cell-to-cell and cell-to-matrix connections [12]. It has been reported that the 3D culture of human ADSCs promotes cell yields, maintains stemness, and represents a promising strategy for cell expansion on industrial levels, with great potential for cell therapy and biotechnology [11,13,14].

In this study, we devised a simple protocol for the purification culture of ADSCs based on a new 3D culture system comprising a self-attached-cell matrix with low fetal bovine serum (FBS) medium in the presence of growth factors. Using this method, we obtained ADSC spheroids via 3D culture of ADSCs. We called these spheroids three-dimensional adipose stem cells (tdASC), and they retained their multi-differentiation capacity after at least ten passages.

To evaluate the potential application of tdASC, we verified whether tdASC has the potential to transdifferentiate into neural progenitor lineage cells. We previously discovered that short-interfering EID3 (siEID3), which is a small interfering RNA of EP300-like inhibitor of differentiation 3 (EID3), has the potential to promote transdifferentiation of mesenchymal stem cells to NPCs [15], and our experiments indicated that siEID3 can partially cause trans-differentiation of adipose-derived stromal cells into NPC-like cells (unpublished data). The major function of the EID gene family is to serve as a P300/CBP suppressor [16] in response to cell differentiation, growth arrest, or apoptosis. The EID protein family includes EID1, EID2, and EID3. EID3 is homologous to a region of EID1, binds to p300/CBP, and acts as an inhibitor of p300/CBP-dependent transcription by direct interaction with nuclear receptors SHP and SRC1 [17].

In this work, we used siEID3 and a variety of common extracellular matrices to identify the optimal combination for trans-differentiation efficiency of tdASC and found that the combination of siEID3 with laminin/poly-d-lysine matrix can efficiently induce tdASC trans-differentiation to neural progenitor-like cells (iNPLCs) in approximately 9 days. These methods provide valuable interdisciplinary tools that can be used to develop ADSCs for research and clinical trials (such as neurological diseases) in the future.

## 2. Materials and Methods

### 2.1. Monolayer Culture of Human ADSCs and Human Neural Stem Cells (NSCs) Culture

All human adipose tissue-derived stromal cells (ADSCs) used in the present study come from freeze isolated cells, isolation of human ADSCs was performed as we previously described [18]. In this study, no vertebrate animals were used, and all of the experiments are carried out at the cellular level.

In the first two or three culture passages using the traditional monolayer culture method, the cells were plated at 200 cells/cm^2^ densities [19] in T75 flasks (Corning, New York, NY, USA) and cultured in ADSCs culture medium DMEM/F12 supplemented with 10% fetal bovine serum (FBS, AusGeneX, Molendinar, Australia) at 37 °C with 5% CO_2_ at saturating humidity. When cells reached about 80~90% confluency, the cells were detached with Accutase (Life Technologies, Eugene, OR, USA) about 3~5 m and the Accutase was inactivated with serum-containing media.

Human neural stem cells (hNSCs, SCC007, Millipore, Billerica, MA, USA) were routinely expanded according to the manufacturer’s protocol. The NSCs were maintained in laminin-coated culture dishes in ReNCell media (Millipore) supplemented with basic fibroblast growth factor (bFGF-2, 20 ng/mL, Peprotech, Rocky Hill, NJ, USA) and epidermal growth factor (EGF, 20 ng/mL, Peprotech). The human NSCs were plated in the dishes at a cell density of 5 × 10^5^/mL. All NSC experiments were carried out between passages 3 and 10.

### 2.2. Three-Dimension Adipose Stem Cells (tdASC) Spheroid Culture

After the ADSC monolayer culture, single-cell suspensions were produced by incubation Accutase reagent (Life Technologies) dissociation for 5 min at 37 °C with gentle shaking and rinsed with 1 × PBS.

These ADSCs were plated in a 3D culture medium at high density (5 × 10^4^ cells/cm^2^) were cultured for more than 5 days, the components of 3D specific-condition culture medium are DMEM/F12 supplemented with 2% fetal bovine serum (FBS, AusGeneX), human basic fibroblast growth factor (bFGF, 5 ng/mL, Peprotech), human epidermal growth factor (EGF, 2 ng/mL, Peprotech), human PDGF (5 ng/mL, Peprotech), heparin (2 µg/mL, Sigma, Mississauga, ON, USA), L-ascorbic acid 2-phosphate sesquimagnesium salt hydrate (50 µg/mL, Sigma), 100 units/mL penicillin, and 100 μg/mL streptomycin. FBS needs to test by batch to batch assays before use to ensure it was sufficient supporting cell proliferation at low concentrations.

**Figure 1:**
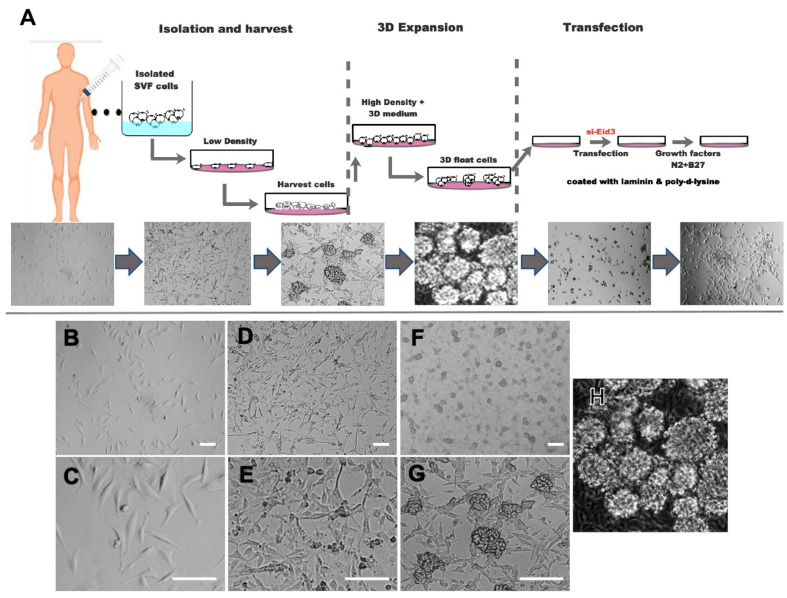
Schematic diagram of the strategy for 3D-cultured adipose stem cell (tdASC) transdifferentiation to neural progenitor-like cells. (**A**). Schematic diagram. tdASC form spheroids and transdifferentiate to iNPLCs. (****B–**H**). Morphological observation of tdASC formation and the characterization of tdASC proliferation. Phase-contrast images of cell expansion at low density (**B**,**C**), high density (**D**,**E**), and tdASC spheres (**F**,**G**). Scale bars: 50 μm. (**H**). Collected tdASC spheroids. Abbreviations: ADSCs, adipose tissue-derived stromal/stem cells; SVF, stromal vascular fraction; siEID3, small interfering E1A-like inhibitor of differentiation 3.

After the ADSCs grew to reach ~90% confluence, some rapidly proliferating cells will form cell spheres, and these spheroids gradually became larger and then they formed semi-suspend spheroids on the attached-ADSCs (Figure 1), we called these three-dimensional ADSC spheroids, that is, the spheroid cells are the three-dimensional adipose stem cells (tdASC).

The medium was replenished with fresh medium every 2 days. The culture procedure is depicted in Figure 1. When more than ten spheroids are formed per square centimeter, primary spheroids were collected by vigorous shaking and centrifugation at 100× *g* for 3 min. tdASC spheroids were dissociated into single tdASC cells with Accutase for 5–8 min followed by another passage or were used for follow-up experiments. When another passage was conducted, the single cells were reseeded into the original flasks where some attached ADSCs remained, these cells formed semi-suspend spheroids again within 3~5 days; the above processes were then repeated for each passage.

### 2.3. Cell Counting Kit-8 (CCK-8) Assays

Cell proliferation was measured using the Cell Counting Kit-8 (CCK-8, Dojindo, Kumamoto, Japan). ADSCs and single tdASC cells (which were dissociated into single cells with Accutase) were plated into a 96-well microplate with 10% FBS DMEM/F12 medium at a density of 1000 cells per well. On days 2, 4, 6, 8, and 10, CCK-8 reagent (10 μL per well) was added to the cells and then incubated at 37 °C for 2 h, then the growth curves of cells were generated using absorbance values detected with a microplate reader (Bio-Rad, Hercules, CA, USA) at 450 nm.

For count the cells, ADSCs and tdASC that were viable and dead were counted using either the Countess^®^ Automated Cell Counter (Invitrogen, Carlsbad, CA, USA) or hemocytometer and using Trypan blue (Invitrogen) exclusion. The duplicate cell counts were averaged to get the final cell number at each time point and the experiment was performed 4~5 independent times.

### 2.4. Proliferation Assays

ADSCs (P1) or tdASC were plated at a density of 8000 cells/cm^2^ in duplicates in a 6 well plate and cells/cm^2^ were quantified at 2, 4, 6, 8, and 10 days, both types of cells were counted when a subconfluence of 80% to 90% was reached. The cells were counted by Countess^®^ and replated at a 1:10 dilution. The obtained average cell number was plotted against the culture time to obtain a growth curve. Using the following formula to calculate the population doubling time (PDT) [20]:

$$\mathrm{PDT}=\mathrm{CT}/(\mathrm{Log}\left(N/N_{0}\right)\times 3.33$$

In the above formula, PDT represents population doublings time; N is the final cell number; N_0_ is the initial number of cells plated; DT is doubling time, and CT is the time in culture.

### 2.5. Alkaline Phosphatase (ALP) Staining and Alkaline Phosphatase Activity

Alkaline phosphatase (ALP) staining, 12,000 cells/cm^2^ were seeded in 12 well plates, after culturing for about 24 h, cells were fixed in 4% PFA (paraformaldehyde, Beyotime, Shanghai, China) for 15 min, and washed twice with PBS, then alkaline phosphatase staining was checking with the 5-bromo-4-chloro-3-indolyl phosphate-nitro blue tetrazolium salt (BCIP/NBT) ALP color development kit (Beyotime) for 30 min, then the images were taken. Data are expressed as a percentage of stained area relative to the control which is defined as 100%.

### 2.6. Immunophenotypic Characterization by Flow Cytometry

The cells were collected by centrifugation and transferred to EP tubes, and cells were suspended in 1% BSA PBS containing 10 µg/mL of the following antibodies: CD29, CD44, CD73, CD90, CD105, CD166, CD34, and CD45, for information on these antibodies, see Table S1. In flow· cytometry analysis, >30,000 events were measured per sample, isotype-stained cells and unstained were used as controls. All data were analyzed by Accuri™ C6 (BD Bioscience, San Jose, CA, USA) and Prism 8 (mac OS version, GraphPad Software Inc., San Diego, CA, USA) software.

### 2.7. siEID3 Transfect and Generation of NPC-Like Cells from tdASC

The transfection procedures are shown in Figure 4A tdASC were dissociated with Accutase (Life Technologies) and plated on 6 well plates (Corning) at a concentration of about 1.3 × 10^5^ cells/cm^2^ in pretreated transfection medium, this transfection medium used was: DMEM/F12 and Neurobasal (1:1, Life Technologies) containing 1% N2 (Life Technologies) and 2% B27 (Life Technologies) supplemented with 5 ng/mL of both epidermal growth factor (PeproTech) and basic fibroblast growth factor (PeproTech) for more than 12 h. Then, the tdASC were transfected with human EID3 siRNA and control siRNA.Targeting EID3 specific siRNAs were 5′-GAATAAGGCTTGATGAAGA-3′ (sense) and 5′-TCTTCATCAAGCCTTATTC-3′ (antisense), and EID3 siRNA control was generated using the nonspecific primers: 5′-AAACGTGACACGTTCGGAGAA-3′ (sense) and 5′-AATTCTCCGAACGTGTCACGT-3′ (antisense), they were synthesized and purified by Sangon Biotech (Shanghai, China), a total amount of 20 nM siEID3 or corresponding control was transfected into cells by INTERFERin^®^ transfection reagent (Polyplus Transfection, Illkirch, France) in accordance following the manufacturer’s protocol. Preliminary experiments show that the transfection efficiency of the FAM-siRNA is at least 90% (Supplementary Figure S2). The medium was removed and changed to the N2B27 induction medium after six hours, and cells were kept cultured at 37 °C with 5% CO_2_ for another 48 h. The N2B27 induction medium is comprised of neurobasal: DMEM/F12 (1:1 ratio), GlutaMAX (Gibco Lab., Grand Island, NY, USA), growth factors EGF and FGF2 (PeproTech), B27 and N2 supplemental factors (Gibco), and antibiotics (penicillin and streptomycin, Gibco). The medium was changed every two days.

After 5 days, the induction medium was replaced with a proliferation medium, which comprised neurobasal DMEM/F12 (1:1 ratio, Life Technologies), FGF2 growth factors 5 ng/mL (PeproTech), B27 supplemental factors (Gibco), and antibiotics (penicillin and streptomycin, Gibco). The cells were cultured for approximately 4 days (3–5 days) and then used for experiments.

### 2.8. Substrates Preparation

To examine different substrates’ effects on NPCs transdifferentiation of tdASC, the treated cell culture dishes (Corning) were coated with the following substrates (all obtained from Sigma-Aldrich except if otherwise mentioned): gelatin, poly-d-lysine, human laminin, type I collagen, and Matrigel (Corning). In brief, tdASC was dissociated with Accutase (Life Technologies) and plated on different matrix tissue culture plates (Corning Inc.) Matrix detail as follows: 0.1% gelatin was prepared with sterile ddH_2_O and was used at 200 μL/cm^2^. Type 1 collagen was used at 0.25~1 mg/cm^2^. Poly-d-lysine was dissolved with sterile ddH_2_O. The poly-d-lysine solution was added to plates at a concentration of 0.1–1 mg/mL and left at room temperature overnight. Laminin was prepared at 10–100 μg/mL in PBS, and laminin was diluted to 1 µg/mL in DPBS, and then 1 mL of solution added to 6 well-plates and keep at 37 °C for 2 h or at 4 °C for 24 h.

For laminin/poly-d-lysine coating as described above, dishes were pretreated with poly-d-lysine at room temperature overnight and then laminin was added at 1 μg/mL in DPBS at 37 °C for 2 h, then the excess substrate was removed and dishes were rinsed with PBS.

### 2.9. Neural Stem Cell Differentiation

For NSC differentiation, the induced cells or spheres were dissociated into single cells, and then the cells were plated at 12,500 cells per cm^2^ in 12 well plates with coverslips in a differentiation medium containing less than 1% FBS. Cells were fixed using 4% paraformaldehyde in PBS and immunostained at 14 days after induction. For three specific lineages (neuron, astrocyte, and oligodendrocyte) differentiation, according to a previously described method [21].

### 2.10. Immunocytochemistry and Confocal Microscopy

Cells were seeded as 1.25 × 10^4^ cells/cm^2^ on coverslips in 12-well plates, and cells were fixed in 4% paraformaldehyde in phosphate-buffered saline (PBS). Permeabilization and blocking were performed in 5% BSA and 0.25% Triton X-100 in PBS for 30 min. Cells were stained with primary antibody at 4 °C overnight. The secondary antibody was applied for 2 h at room temperature. Sub-cellular images were determined using a confocal imaging system (CLSM, TCS SP5 II, Leica, Wetzlar, Germany).

Cells were fixed in 4% paraformaldehyde in PBS. Immunocytochemistry was carried out using standard protocols. Fluorescence labeled secondary antibodies (donkey anti-rabbit secondary antibody Alexa Fluor 488 (1:1000), donkey anti-mouse IgG Alexa Fluor 568 (1:1000)) were then applied. Cell nuclei were counterstained with 4,6-diamidino-2-phenylindole (DAPI).

### 2.11. RNA Extraction, Quantitative Real-Time RT-PCR Analysis

RNA was isolated from cells using TRIzol (Invitrogen) according to the manufacturer’s protocol. Total cellular RNA was extracted from ADSCs, tdASC, and iNPCLs using TRIzol followed by treatment with RNase-free DNase according to the manufacturer’s protocol. To determine the expression levels of mRNA, total RNA was reverse transcribed with a PrimeScript^®^ RT Reagent Kit (TaKaRa BIO, Shiga, Japan). Approximately 500 ng of total RNA was used for the first-strand cDNA synthesis. Quantitative real-time RT-PCR was carried out using Applied Biosystems^®^ ViiA™ 7 System and subsequently amplified using the SYBR Green PCR Master Mix (TaKaRa) and 0.5 µM each of the sense and antisense primers. After amplification, the melting curves of the RT-PCR products were acquired to demonstrate product specificity. Results are expressed relative to the housekeeping gene GAPDH. Primer sequences are summarized in Table S2.

### 2.12. Statistical Analysis

The SPSS statistical software package (Chicago, IL, USA) was used for statistical analysis. All experiments were performed at least in triplicates. Data were presented as mean ± standard deviation (SD). Comparisons were accomplished by one-way analysis of variance (ANOVA) with LSD post-hoc test or student’s *t*-test. A statistical significance was defined as *p* < 0.05.

## 3. Results

### 3.1. Three-Dimensional ASC Spheroid (tdASC) Expansion and Differentiation

Several studies have explored culturing 3D cell spheroids of ADSCs [8,21], and these techniques have been further improved by using various coating agents and materials. In this study, we established a novel and economical semi-suspended method for culturing 3D spheroids of ADSCs (Figure 1A, and more details are given in the Materials and Methods). Semi-suspended spheroids were formed on adherent ADSCs cells attached to plastic plates, with the ADSC-derived self-feeder cell layer playing a role in supporting and joining the semi-suspended tdASC spheroids (Figure 1B–H). In addition, we cultured different batches of ADSC according to this method, these ADSCs are also formed three-dimensional spheres on the ADSC-derived self-feeder cell layer (Supplementary Figure S1).

**Figure 2:**
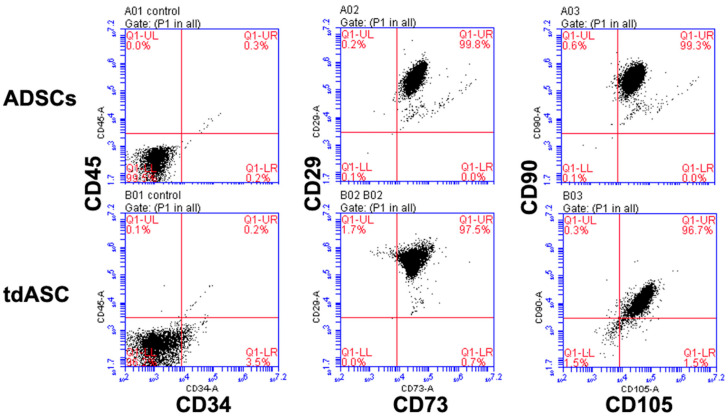
Immunophenotyping of ADSCs and tdASC by FCM analysis. P3–4 ADSCs and tdASC were positive for MSC markers CD29, CD73, CD105, CD90, and negative for CD34 and CD45.

Flow cytometry (FCM) showed that expression of surface markers, such as CD29, CD73, CD90, and CD105, recommended by the ISCT was greater than 98%, confirming the mesenchymal stem cell characteristics of the cells (Figure 2). Furthermore, expression of CD34 and CD45 surface markers in the isolated ADSCs was less than 5%, suggesting that no endothelial or hematopoietic lineage cells were present (Figure 2). These results show that the ADSCs and tdASC met the minimal ISCT criteria

### 3.2. tdASC Cell Proliferation and Population Senescence Analysis

**Figure 3:**
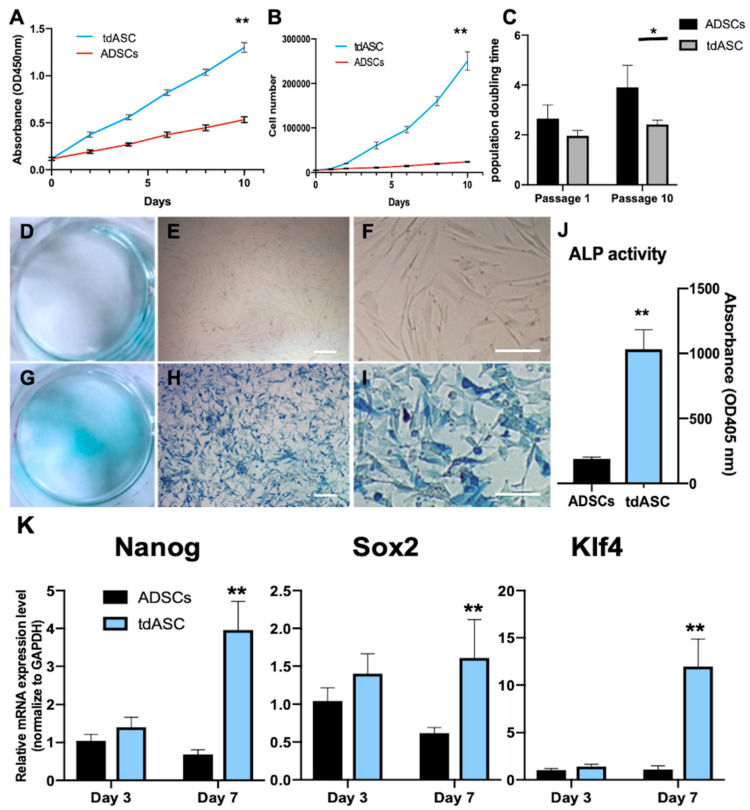
Characterization of tdASC and ADSC proliferation and senescence. (**A**). Cell counting kit 8 (CKK-8) assay. tdASC cell lines showed a significant increase in proliferation; *n* = 5; ** *p* < 0.01. (**B**). Cell counting plot for tdASC and ADSCs; *n* = 5; ** *p* < 0.01. (**C**). Population doubling time (PDT) plot of tdASC and ADSC self-renewing culture (for the formula, see materials and methods); *n* = 5; * *p* < 0.05. (**D**–**I**). At passage 10, ADSCs (**D**–**F**) and tdASC (**G**–**I**) were fixed and stained with a BCIP/NBT assay kit. Scale bars: 20 μm. (**J**). Quantification of ALP-positive tdASC and ADSCs at passage 10. *n* = 5; ** *p* < 0.01; data are presented as the mean SEM. (**K**). qRT-PCR measurements for stem cell marker genes (Nanog, Sox2, and Klf4) in ADSCs and tdASC. *n* = 3; ** *p* < 0.01; data are presented as the mean SEM.

To explore tdASC proliferation characteristics, the collected tdASC spheroids were passaged using 2D culture methods, namely, tdASC were dissociated with Accutase and passaged at 5000 cells/cm^2^ in 6-well plates. ADSCs served as the control group. Next, the tdASC were cultured in DMEM/F12 supplemented with 10% FBS for 10 days; CCK-8 assays were carried out every 2 days, and total cell counts were performed at the same time (Figure 3A,B). At the end of 10 days, the absorbance of the tdASC group and the control group showed significant differences (1.30 ± 0.052 and 0.533 ± 0.051, *p* < 0.05, Figure 3A); the number of tdASC and control cells was 2.5 × 10^5^ ± 1.05 × 10^4^ and 2.4 × 10^4^ ± 882, respectively (Figure 3B).

Then, the tdASC and ADSC population doubling times (PDTs) were calculated. At passage 1, the tdASC PDT was 2.66 ± 0.32 days compared to 2.0 ± 0.12 days for the expanded ADSCs, and there was no difference at passage 1. At passage 10, the tdASC PDT was 2.42 ± 0.10 compared to 3.91 ± 0.51 for the control group, and there was a significant difference (*p* < 0.05, Figure 3C).

Alkaline phosphatase (ALP) activity is a well-known biomarker for stemness. To assess any difference in stemness between the tdASC and ADSC groups, the cells were analyzed with an alkaline phosphatase kit and stained. There was a much higher number of ALP-positive tdASC than ADSCs at passage 10 (Figure 3D–I). Based on ALP activity quantification, ALP activity in tdASC was significantly higher than that in ADSCs at passage 10, i.e., 2936% ± 108% (*p* < 0.01; Figure 3J).

### 3.3. tdASC Stemness Gene Expression

The relative mRNA expression levels of the transcription factors Nanog, Sox2, and Klf4, which are related to stemness, were analyzed by qRT-PCR. On 3 day, there was no difference in expression of Nanog, Sox2, and Klf4, between tdASC and ADSCs. However, after 3D culture for 7 days, expression of Nanog, Sox2, and Klf4 was significantly upregulated in the tdASC spheroids, which increased by 3.1 times, 1.8 times, and 6.3 times, respectively, compared with control ADSCs (Figure 3K).

### 3.4. Identification of the Optimal Matrix for tdASC Induction

**Figure 4:**
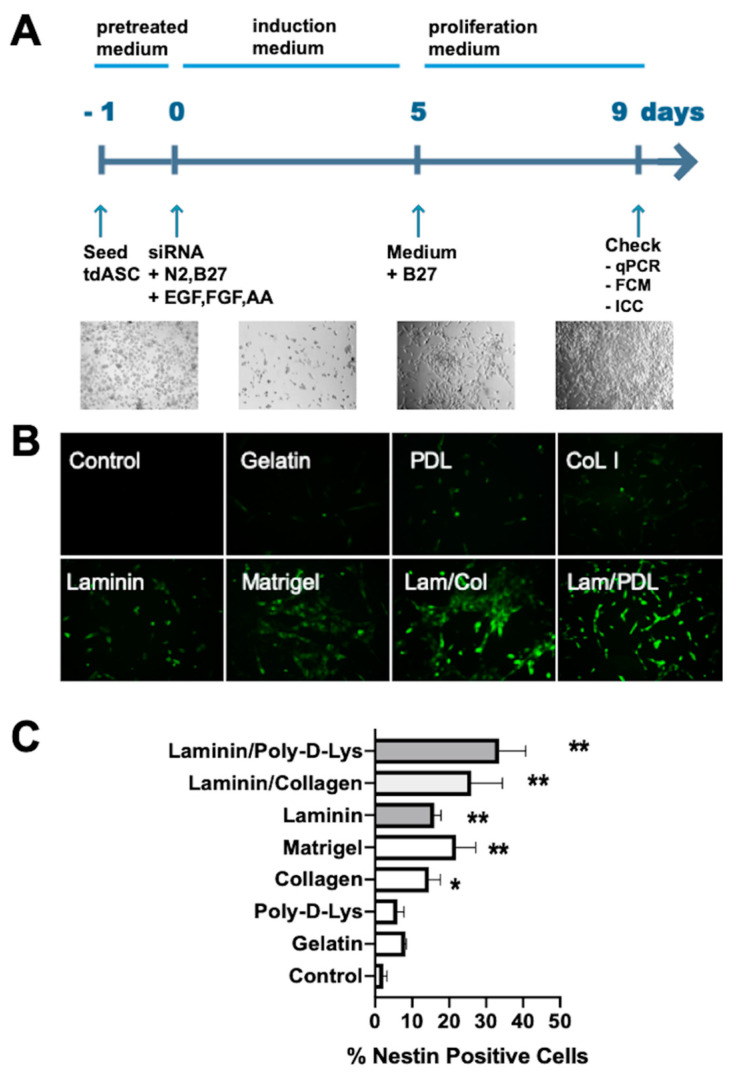
Matrix screen for tdASC-derived NESTIN+ neural progenitor-like cells (iNPLCs). (**A**). Experimental scheme of the generation, expansion, and verification of induced neural progenitor-like cells. (**B**). A panel of images showing representative regions of NESTIN+ cells expanded on gelatin, poly-d-lysine (PDL), collagen I (Col), laminin (LM), Matrigel, LM/Col, and LM/PDL substrates on day 3. (**C**). Values are expressed as the number of NESTIN+ cells per field. *n* = 6; * *p* < 0.05; ** *p* < 0.01; data compared to the control group; data are presented as the mean SEM.

The above results indicated that tdASC has strong stemness properties. Our previous and some published studies have shown that siRNA in combination with specific matrices (such as Matrigel) promote the transdifferentiation of adult stem cells to neural cells or NSCs [15,21,22,23,24,25]. We discovered in previous NPC induction studies that Matrigel can improve NPC induction efficiency (unpublished data). However, Matrigel also has some insurmountable disadvantages, such as varying compositions of biopolymers, batch effects, and a relatively expensive cost. Therefore, we aimed to identify a defined type of matrix that works better than Matrigel, and we combined siEID3 with different matrices to cause tdASC to transdifferentiate to NPCs, Figure 4A shows the generation scheme.

tdASC were plated in cell culture dishes coated with gelatin, laminin, collagen I (Col I), Matrigel, laminin/collagen I (Lam/Col), or laminin/poly-d-lysine (Lam/PDL). After we seeded the tdASC and ADSCs on these substrates, we conducted the transfection process described above followed by ICC experiments. NESTIN+ cells were quantified over 3 days in randomly selected fields. The number of NESTIN+ cells per unit area, ranging from low to high (Figure 4B), was 5.6 ± 0.5%, 7.1 ± 0.8%, 12.0 ± 1.4%, 16.8 ± 0.8%, 22.3 ± 1.5%, 27.3 ± 2.3%, and 36.4 ± 3.0% for gelatin, laminin, collagen I (Col I), Matrigel, laminin/collagen, and laminin/poly-d-lysine, respectively (Figure 4C). The greatest number of NESTIN+ cells was obtained in the laminin/poly-d-lysine matrix; therefore, in subsequent transdifferentiation experiments, we used laminin/poly-d-lysine as the default matrix for transdifferentiation.

### 3.5. Generation and Characterization of iNPLCs

Based on the above results and related experiments, we devised an induced neural progenitor-like cells (iNPLCs) generation scheme, as shown in Figure 4A: in short, tdASC spheroids were dissociated into single cells by Accutase treatment, and the cells were seeded on the laminin/poly-d-lysine matrix. On the next day, the tdASC was transfected with small interfering EID3 (siEID3), and the cells were cultured with the induction medium. After 5 days of culture in the trans-differentiation medium, a marked change in the appearance of the differentiated tdASC was observed. After 9 days, the expression of neural stem cell markers was assessed at mRNA and protein levels using qRT-PCR, FCM, and ICC.

In subsequent experiments, we confirmed the effect of siEID3 transfection plus laminin/poly-d-lysine on trans-differentiation. For this, experiments were carried out with the following four groups: negative siRNA control with laminin/poly-d-lysine, siEID3-Martigel, NSC group (positive control, NSCs cultured on laminin matrix), and control group (negative control, ADSCs).

**Figure 5:**
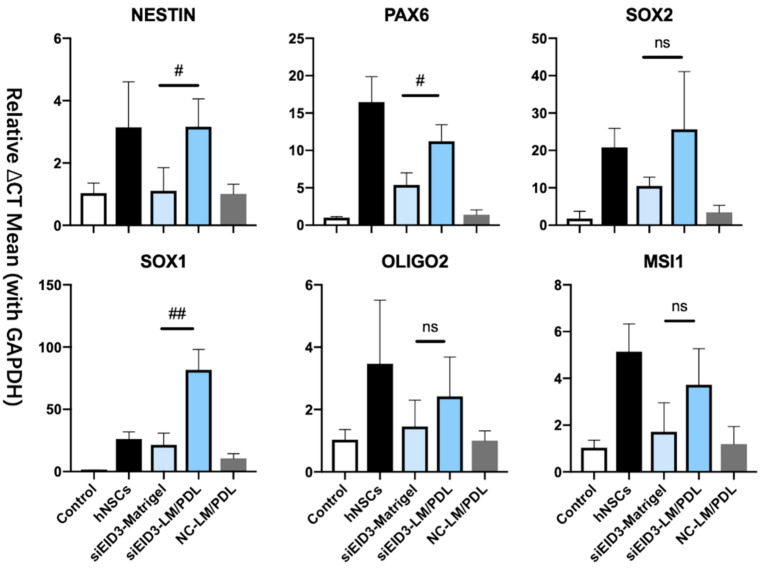
Characterization of iNPLCs differentiated from tdASC by different methods. Real-time qRT-PCR of the iNPLC and early NSC markers NESTIN, PAX6, SOX2, SOX1, OLIGO2, and MSI1 (Musashi-1) in each experimental group. Control, negative siRNA control; hNSCs, commercial human NSCs; LM/PDL, laminin/poly-d-lysine. The mRNA level of each group was normalized to the corresponding GAPDH level. The bars represent the mean ± SEM of at least three independent experiments. *n* = 3; # *P* < 0.05; ## *P* < 0.01; data compared to the control group (hADSCs); ns, not significant; data are presented as the mean SEM.

First, real-time qRT-PCR showed that the expression levels of markers, such as NESTIN, PAX6, SOX2, SOX1, OLIGO2, and Musashi-1 (MSI1), were significantly increased in the tdASC in the laminin/PDL group compared with those in the ADSC control group (Figure 5). Furthermore, as Matrigel is the most commonly used base matrix for NSCs [26,27], we examined the difference between laminin/PDL and Matrigel. We found that the mRNA expression level of main NSC markers, such as PAX6, NESTIN, and SOX1, was significantly higher in the laminin/PDL group than in the Matrigel group. The expression level of SOX2, OLIGO2, and MSI1 did not differ significantly between the laminin/PDL and Matrigel groups. This result shows that the effect of LM/PDL matrix on transdifferentiation is probably better than Matrigel.

**Figure 6:**
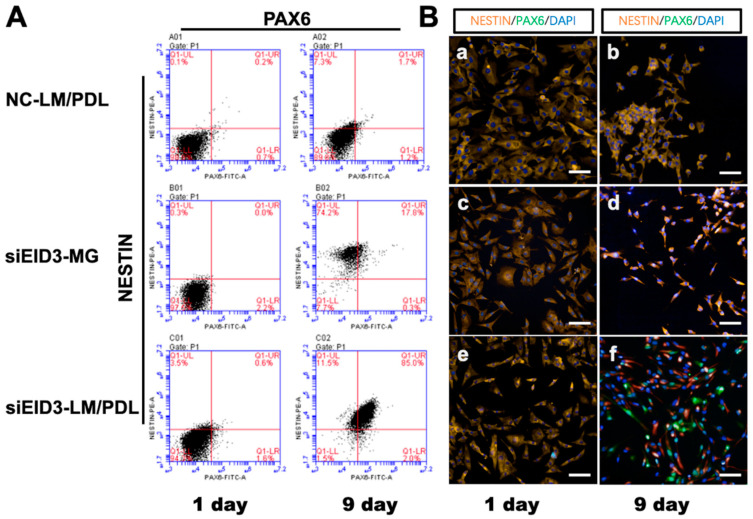
Immunophenotyping changes after tdASC induction. (**A**). FCM analysis of NSC markers NESTIN and PAX6 after tdASC induction was carried out with negative control, siEID3-Matrigel, and siEID3-LM/PDL groups on days 1 and 9. Representative images for each group and differentiation condition are shown. NC, negative siRNA control; LM/PDL, laminin/poly-d-lysine. (**B**). Immunocytochemistry analysis of NSC markers NESTIN and PAX6 after tdASC induction was carried out with negative control, siEID3-MG, and siEID3-LM/PDL groups on days 1 and 9. (**a**,**b**) Negative control groups, (**c**,**d**) siEID3 on Matrigel matrix, and (**e**,**f**) siEID3 on laminin/poly-d-lysine matrix were stained for PAX6 (green), NESTIN (red), and DAPI (blue).Scale bars: 50 μm. Representative images for each group and differentiation condition are shown. NC, negative siRNA control; LM/PDL, laminin/poly-d-lysine; MG, Matrigel.

Next, expression of NESTIN and PAX6, which are two NSC markers, at the protein level was assessed after tdASC trans-differentiation to NPCLs on the laminin/poly-d-lysine matrix. The FCM study showed that NESTIN (at 96%) and PAX6 (at 87%) were highly expressed in the transdifferentiated group compared to the control group at 9 days post-plating (Figure 6A).

Immunohistochemical staining of NSC marker proteins (NESTIN and PAX6) in the reprogrammed tdASC was investigated. The expression level of NESTIN was obviously increased when using laminin/poly-d-lysine, and NESTIN and PAX6 were highly expressed in the transdifferentiated group compared to the control group. The results are presented in Figure 6B. To further determine the differentiation ability of the tdASC-derived induced neural progenitor-like cells (iNPLCs), we carried out neural lineage differentiation assays, such as for differentiation into neurons (Supplementary Figure S3D), astrocytes (Supplementary Figure S3E), and oligodendrocytes (Supplementary Figure S3F); tdASC was used as the control group (Supplementary Figure S3A–C).

In short, iNPLCs generated from tdASC were found to be multipotent, similar to neural stem cells.

## 4. Discussion

Adipose-derived stem cells (ADSCs) have become a prospective stem cell source for clinical cell-based therapy [28,29]. Compared to other sources of adult stem cells, ADSCs are now the preferred choice due to their accessibility, abundance, and easy collection procedure [6]. Indeed, it is for these reasons that ADSCs are currently used extensively in a wide range of therapeutic applications in clinical trials [28,30], but most of these applications mainly make use of the paracrine and nutritional functions of ADSCs. Currently, there are very few cases in which ADSCs are directly transdifferentiated to neural stem/progenitor cells (NSCs/NPCs) and then used in the clinic [31].

One of the reasons for this is that ADSCs comprise a group of heterogeneous subpopulations that have different proliferation and differentiation capabilities in vitro and in vivo [9]. This heterogeneity may be attributed to donor variability, various isolation protocols, or a lack of suitable markers for cell purification. There are several optimized methods to address these issues: low plating density [32], negative and positive selection based on specific surface marker expression [33,34], and centrifugation with a Percoll gradient [5]. Most of these methods are inconvenient, costly, and have poor reliability.

In addition, these methods do not always lead to ADSC purity or long-term cultivation, and they basically did not solve the problem of replicative senescence of different donor ADSC varied greatly. According to previous studies and our experimental experience, the passages of ADSCs of different donors have huge differences, some cultures stopped proliferating as early as after three or four passages (cumulative PDs were 6~10) [35,36]. Under our culture conditions, tdASC of three donors were all capable of proliferating for at least 10 passages (approximately 6–7 population doublings per passage). Furthermore, this tdASC had passed through the above multiple passages, and no assay showed that tdASC had signs of senescence. We are ready to further extending the cell culture time and increasing the passage number in order to obtain more evidence.

There is existing evidence that forced stem cell suspension can affect stem cell proliferation and differentiation capabilities; that is, the cell suspension (3D) culture method enhances cell stemness properties and decreases cell differentiation characteristics [37]. Sphere formation may be one of the significant characteristics of robustly proliferative and early-stage stem cells, such as embryonic stem cells forming spheres or colonies on the feeder or neural stem cells forming neurospheres [38]. In our three-dimensional adipose stem cell (tdASC) culture scheme (Figure 1), we take advantage of the inherent proliferative heterogeneity properties of ADSCs, allowing a small subset of highly proliferative subpopulations to form cell spheroids on specific matrix scaffolds; the special matrix is formed by a subset of plastic-adherent ADSCs. Since the number of cells is very small compared to the whole ADSC population, there has been no effective way to screen these spheroid-forming cells from the ADSC population, though our method provides an effective solution. As this method of cell culture does not require other exogenous extracellular matrices or scaffolds, the scheme is simple and economical. In short, tdASC spheroid formation in our developed method occurs due to the following reasons: (1) highly proliferative cells will grow as spheroids, and (2) scaffolds are formed by ADSCs themselves, providing not only a cell-matrix but also supplying nutrition, signaling, and other features, such as growth factors and exosomes.

For the 3D cell culture of mesenchymal stem cells (MSCs), the most common culture method is scaffold-free 3D culture, which uses ultralow attachment culture flasks [10]. With this method, all the cells are forced to form cell spheroids because they cannot attach to the plastic dish surface; the spheroids form entirely by adherent cell aggregation after being forced into suspension, unlike neurospheres, which form via rapid self-organization after cell proliferation. For this reason, both stem cells and differentiated cells exist among the cell spheroids formed by 3D cell culture. Additionally, when the cell spheroid size increases slightly in ultralow attachment cell sphere culture, the core of the 3D sphere gradually turns black, and the cells in the center of the spheroid die; this condition leads to a significant reduction in cell numbers and shortened cell passage times. With our newly developed method, the cell spheroids are composed of cells with a strong proliferation capability. In contrast to other 3D culture protocols, our semi-suspended 3D method avoids the use of any artificial physical or chemical method to remove specific subpopulations or senescent cells, resulting in a high yield of ASCs with strong stemness that is maintained for a long time.

In current clinical research, ADSCs are applied for the treatment of neurological diseases, and high-quality or clinical-grade NSCs/NPCs are a prerequisite for the clinical treatment of neurological diseases [39,40]. However, due to the scarcity of endogenous NSCs/NPCs, the trans-differentiation of adult stem cells to NSCs/NPCs has become one of the most important alternatives. We know that the specific matrix components are very important for neural stem cell reprogramming or trans-differentiation [6,7]. The extracellular matrix (ECM) is a mixture of biological macromolecules, usually large glycoproteins, including gelatin, laminin, fibronectin, and collagen assembled into fibers or other complex macromolecular arrays. To trans-differentiate ADSCs to neural progenitor cells, we screened multiple commonly used biological matrices and discovered that the use of the laminin/poly-d-lysine matrix with siEID3 markedly improved the efficiency of cell transdifferentiation and shortened the trans-differentiation period.

Furthermore, we discovered that siEID3 possesses enough potential to trans-differentiate tdASC into NPLCs, the possible reasons are: the major function of EID3 is to serve as a P300/CBP suppressor in response to cell differentiation [16]. P300 is a HAT histone acetyltransferase, and P300 plays a critical role as a transcriptional coactivator and in histone acetylation and open-conformation chromatin [15]. In this study, siEID3 inhibited EID3 and maintained the P300 activation state by keeping chromatin open and promoting trans-differentiation. Moreover, laminin/PDL can interact with integrins a6 and regulate differentiation [41] through the MAPK/ERK signaling pathway [42]. Consequently, siEID3 with the laminin/PDL matrix improved the efficiency of neural progenitor cell trans-differentiation.

These findings may facilitate studies of mesenchymal stem cell trans-differentiation. There is a large obstacle to the use of MSCs transdifferentiated into stem cells, which makes it impossible to obtain stable stem cell populations with consistent properties. The method developed in this study utilizes proliferative and self-organizing substrates, which can essentially be obtained without exogenous culture. Besides, we applied this method to successfully and rapidly trans-differentiate ADSCs to iNPCLs in vitro. Next, we plan to verify the proliferation and differentiation capabilities of tdASC-transdifferentiated iNPCLs in mice and primates.

In short, the results of this study provide a set of feasible methods for culturing and screening ADSCs with relatively uniform properties and a strong proliferation capacity and demonstrate the ability of tdASC to trans-differentiate to iNPCLs, which have clinical application potential for neurological disease treatment.

## Data Availability

Some or all data, models, or used during the study are available from the corresponding author by request.

## Supplementary Materials

The following are available online at https://www.mdpi.com/2073-4409/10/3/493/s1, Figure S1: Morphological observation of 3D-cultured adipose stem cell (tdASC) formation, Figure S2: siRNA transfection efficiency in control (ADSCs) and tdASC cells at 48 h post-transfection, Figure S3: Immunocytochemistry images of iNPLC differentiation into three neural cell lines, Table S1: List of antibodies, Table S2: List of primers for qRT-PCR assays.
