# Supplementary material for: Based on a Self-Feeder Layer, a Novel 3D Culture Model of Human ADSCs Facilitates Trans-Differentiation of the Spheroid Cells into Neural Progenitor-Like Cells Using siEID3 with a Laminin/Poly-d-lysine Matrix
Source: Cells. 2021 Feb 25;10(3):493. doi: 10.3390/cells10030493 (PMC7996540; doi:10.3390/cells10030493)
Supplement: Supplementary file 1 [file cells-10-00493-s001.zip › S1-Figure-supplement.docx]

Fig. S1


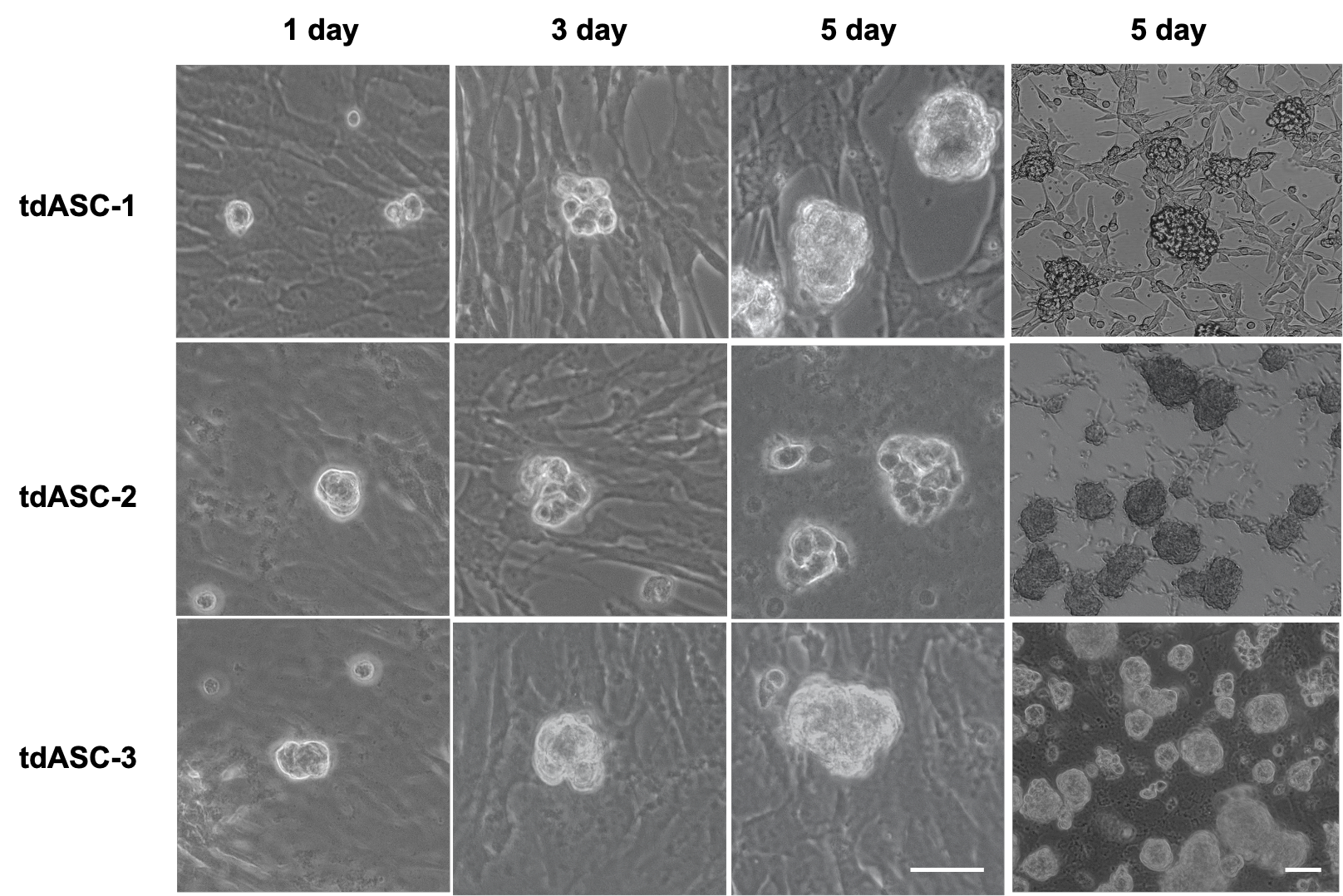


Figure S1. Morphological observation of 3D-cultured adipose stem cell (tdASC) formation.

Morphological observation of three different human ADSCs cell lines formed tdASC on 1 day, 3 day, and 5 day. Scale bars: 50 µm. Origin of ASC in abdominal subcutaneous fat, the age and gender information of donors of tdASC: tdASC-1, 25, female; tdASC-2, 31, male; tdASC-3, 29, male.

Fig. S2


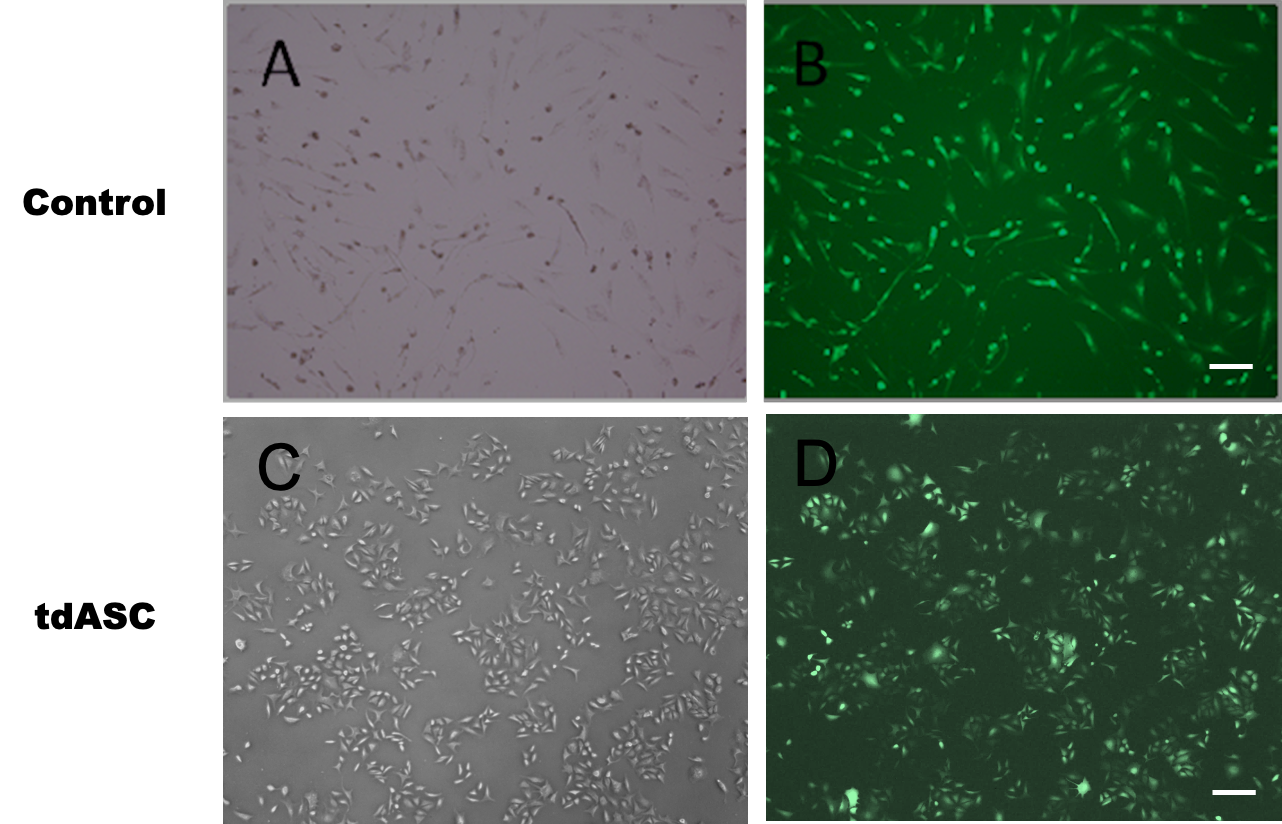


Figure S2. siRNA transfection efficiency in control (ADSCs) and tdASC cells at 48 h post-transfection.

Phase-contrast micrographs (A, C) and fluorescence (B, D) of ADSCs (A, B) and tdASC (C, D) cells at 48 h post-transfection. (w/w). Scale bar = 50 μm.

Fig. S3


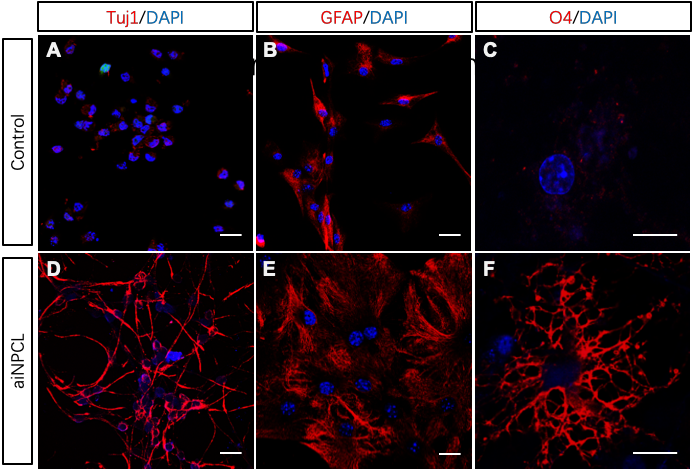


Figure S3. Immunocytochemistry images of iNPLC differentiation into three neural cell lines.

Control ADSCs cells (A, B, C) and iNPLCs (D, E, F) differentiated into neurons with stained for Tuj1 (A, D), oligodendrocytes stained for GFAP (B, E), and astrocytes stained for O4 (C, F) and with DAPI (blue) on day 14. Scale bars: 20 µm.
